# Assessing schema modes for eating disorders and their association with personality traits: validation of the English version of the short form of the schema modes inventory for eating disorders (EN-SMI-ED-SF)

**DOI:** 10.1186/s40337-025-01446-0

**Published:** 2025-11-11

**Authors:** Susan Simpson, Alessandro Alberto Rossi, Stefania Mannarini, Dorothy Tait, Gianluca Castelnuovo, Giada Pietrabissa

**Affiliations:** 1 NHS Forth Valley Eating Disorder Service, Larbert, Scotland, UK; 2 Department of Justice and Society, University of South Australia, Adelaide, Australia; 3 Department of Philosophy, Sociology, Education, and Applied Psychology, Section of Applied Psychology, University of Padova, Padova, Italy; 4 Center for intervention and Research on Family studies-CIRF, Department of Philosophy, Sociology, Education, and Applied Psychology, Section of Applied Psychology, University of Padova, Padova, Italy; 5 Child and Adolescent Mental Health Service, NHS Western Isles, Glasgow, Scotland; 6 Psychology Research Laboratory, Ospedale San Giuseppe, IRCCS Istituto Auxologico Italiano, Verbania, Italy; 7 Department of Psychology, Catholic University of Milan, Milan, Italy

**Keywords:** Schema modes, Schema modes inventory, Eating disorders, Early maladaptive schemas, Schema therapy, Eating behaviors, Personality traits

## Abstract

**Background:**

Schema Therapy is an integrative psychotherapy model with a growing evidence base in the successful treatment of eating disorders (ED). To specifically assess schema modes in ED, the Schema Mode Inventory for Eating Disorders – Short Form (SMI-ED-SF) was developed but its English adaptation is still lacking.

**Objective:**

The aim of this study is to evaluate the psychometric properties of the English version of the SMI-ED-SF (EN-SMI-ED-SF) within a community sample and to explore its relationship with ED conditions and personality traits.

**Method:**

An observational design was used, recruiting participants from the general population and specialized clinics for ED treatment. A confirmatory factor analysis was performed to test its factorial structure. A multivariate analysis of variance was conducted to ascertain differences between ED conditions on the dimensions of the EN-SMI-ED-SF. Lastly, the relationship between schema modes and personality traits was explored.

**Results:**

The EN-SMI-ED-SF demonstrated excellent fit indices, with robust validity and reliability for measurement of schema modes. Maladaptive schema modes were systematically higher in ED compared to non-ED individuals, and adaptive schema modes were lower in ED groups compared to non-ED individuals. Whereas maladaptive schema modes showed the strongest positive correlations with neuroticism, adaptive modes were strongly correlated with psychological flexibility.

**Discussion:**

The EN-SMI-ED-SF demonstrated robust psychometric properties, indicating its validity and reliability for assessment of schema modes. The results provide strong evidence for the role of schema modes in ED pathology, and their association with key personality correlates and the protective function of psychological flexibility.

**Supplementary Information:**

The online version contains supplementary material available at 10.1186/s40337-025-01446-0.

## Abstract (plain language summary)

Schema Therapy is an emerging therapy model in the treatment of eating disorders. The short Schema Mode Inventory for Eating Disorders was adapted to measure the ‘modes’, or cognitive-emotional self-states, as they apply to individuals with eating disorders. This study explored the psychometric properties of this measure in a community sample. The association between schema modes and specific eating disorder conditions and personality traits was investigated. The Schema Mode Inventory for Eating Disorders was found to be a reliable and valid measure. Maladaptive schema modes were higher for people with eating disorders compared with those with no eating disorder. Demanding and Punitive Critic Modes were significantly higher amongst participants with an eating disorder, particularly those with Anorexia Nervosa, highlighting their strong adherence to unrealistic internalized standards and self-criticism. The Eating Disorder Overcontroller, which manifests as body-focused over-control, was strongest in Anorexia and Bulimia Nervosa. Detached Protector and Detached Self Soother were higher for participants with binge eating. Maladaptive schema modes were strongly associated with neuroticism. Adaptive modes were associated with greater psychological flexibility, indicating the value of strengthening the Healthy Adult and Happy child modes within treatments for eating disorders to facilitate adaptive self-regulation of emotionality and psychological flexibility.

**Supplementary Information:**

The online version contains supplementary material available at 10.1186/s40337-025-01446-0.

## Introduction

### Schema therapy and the schema mode model

Schema therapy (ST) is an integrative psychotherapy model developed by Jeffrey Young [1], originally aimed at treating complex and longstanding psychological difficulties, including personality disorders (PDs). ST extends cognitive-behavioral therapy (CBT) by incorporating elements from attachment theory, psychodynamic approaches, and emotion-focused therapy. At its core, ST posits that early maladaptive schemas (EMS)—broad and pervasive cognitive-affective patterns—develop in response to unmet core emotional needs during childhood and shape an individual’s perceptions, relationships, and behaviours across the lifespan [2–4].

One of ST’s distinguishing features is its emphasis on experiential techniques to access and modify these schemas, alongside the use of the therapeutic relationship as a corrective emotional experience. A key element in ST is the concept of schema modes—transient emotional-cognitive states that emerge in response to specific triggers and guide behavioural responses. Unlike the more stable EMS, schema modes are context-dependent and can shift rapidly, resulting in sudden changes in affect, cognition, and behaviour [1, 5]. They represent the here-and-now manifestations of latent EMS traits and are pivotal to understanding clients’ fluctuating presentations.

Schema modes are grouped into four broad categories: Child Modes (e.g., Vulnerable Child, Angry Child); *Critic (Parent) Modes* (e.g., Punitive Critic, Demanding Critic); *Coping Modes* (e.g., Detached Protector, Compliant Surrenderer, Overcontroller); *Healthy Adult Mode.*

Each mode reflects specific emotional-somatic-cognitive patterns that can either support well-being or reinforce dysfunction. Whilst coping modes often emerge as understandable responses to developmental adversity—such as maltreatment, neglect, abuse [6–8], or overprotection [9–11]—they tend to become self-perpetuating over time, resulting in further emotional pain and relational isolation.

Furthermore, less adaptive modes may arise when the caregiving environment is misaligned with a child’s temperament (e.g., high sensitivity, perfectionism, neuroticism) [12, 13]. According to differential susceptibility theory, the trait of environmental sensitivity heightens responsiveness to both negative and positive caregiving environments, meaning that attuned parenting may buffer risk or confer resilience, whereas misattuned parenting may amplify vulnerability [14].

In contrast to the coping modes, the *Healthy Adult Mode* represents an integrative and adaptive self-state, capable of regulating emotions and meeting interpersonal and emotional needs [15]. A central therapeutic goal in ST is to foster this Healthy Adult functioning while reducing reliance on maladaptive coping modes and EMS.

### Assessment of schema modes

Given the fluctuating and multifaceted nature of modes, accurate assessment is essential for case conceptualisation and treatment planning. Schema modes vary in intensity and prominence, and their systematic evaluation helps clinicians to identify patterns that limit well-being and to develop personalised interventions [16].

To support this, the Schema Mode Inventory (SMI; 124 items) [17] and its short form (SMI-SF; 118 items) [16] are reliable and valid tools. Initially developed to assess schema modes in individuals with PDs, the SMI comprises 14 factors: *Child Modes*: Vulnerable Child, Angry Child, Enraged Child, Impulsive Child, Undisciplined Child; *Coping Modes*: Compliant Surrender, Detached Protector, Detached Self-Soother, Self-Aggrandiser, Overcompensator; *Critic Modes*: Punitive Critic, Demanding Critic; *Healthy Adult.*

### Schema modes and eating disorders

There is a growing recognition of the relevance of schema modes in populations beyond PD, particularly among individuals with complex or chronic eating disorders (EDs) [5, 16, 18–22]. Elevated rates of perfectionism and neuroticism—traits strongly linked with both EMS and coping modes—are well-documented in ED populations [23–26]. These traits may both predispose individuals to disordered eating and be intensified by it. However, it is essential to distinguish between enduring personality traits and characteristics shaped by the psychological toll of a prolonged ED.

Research supports the utility of transdiagnostic, dimensional frameworks to understand personality-related mechanisms in ED, moving beyond categorical diagnoses [27, 28]. One such approach maps EDs onto three personality styles—overcontrolled, undercontrolled, and resilient. These styles appear to better explain symptom severity and treatment engagement than diagnostic category alone [29–32]. Traits like effortful control, perfectionism, emotional regulation, and identity integration influence recovery trajectories [33–35], in part because individuals may rely on disordered eating behaviours to compensate for underdeveloped traits—e.g., using restriction to generate identity, control, or emotional regulation.

Moreover, these traits influence therapy engagement, willingness to complete homework tasks, and capacity for behavioural change [36]. They map closely onto ST constructs: EMS and schema modes that impose harsh standards and drive maladaptive coping.

### Schema modes as mechanisms linking personality and eds

Research supports the mediating role of EMS and schema modes in the relationship between caregiving experiences and ED symptoms [37, 38]. For example, someone with Bulimia Nervosa may develop an Abandonment schema through inconsistent caregiving, which activates the Vulnerable Child mode and may lead to binge eating via the Detached Self-Soother mode. Preliminary evidence supports a sequential model: perceived parenting → temperament → EMS severity → ED symptoms [39].

Compared to healthy controls, individuals with EDs score significantly higher across most modes (except Bully and Self-Aggrandiser modes) [36, 39]. Modes appear to sustain symptom severity by reinforcing rigid, distress-maintaining self-states.

### The eating disorder overcontroller (EDO) and other key modes

The Eating Disorder Overcontroller (EDO) mode—specific to ED presentations—describes overcompensatory behaviours such as dietary restriction, excessive exercise, and preoccupation with body image [36, 37, 40]. It functions to promote perceived mastery, control, and safety by suppressing vulnerability. It overlaps with the Perfectionistic Overcontroller mode (prominent in OCPD), but is characterised in ED by a stronger pursuit of purity, denial of needs, and emotional highs from “triumphing” over bodily urges [41–43].

Other central modes include: Helpless Surrenderer: passive distress communication, reliance on others, withdrawal, linked to abandonment, subjugation, and emotional deprivation schemas [44, 45]; Vulnerable Child: emotional states rooted in unmet needs for safety, love, and play [46]; often overshadowed by critic or protector modes; Punitive Critic: self-loathing, harsh internal demands; Detached Protector: emotional numbing, avoidance, often linked to binge eating or rigid restraint; Compliant Surrenderer: acquiescence to external demands at one’s own expense; Perfectionistic Overcontroller and EDO: drive for achievement through bodily control, hyperautonomy, and denial of emotional needs [47, 48].

The Angry Child may also surface, using eating behaviours as a form of silent protest or rebellion. Overcontrol traits—perfectionism, rigidity, compulsivity—are most prevalent in Anorexia Nervosa, aligning with EDO mode. Conversely, traits of dysregulation and impulsivity—mapping onto Impulsive/Vulnerable/Angry Child and Detached Self-Soother—are more evident in Bulimia Nervosa and BPD [49].

### Mode sequences in eating disorders

ST highlights mode sequences, where one schema or mode triggers another [15, 36]. For instance, a patient with Anorexia may begin in Compliant Surrenderer mode, seeming cooperative. When weight gain is imminent, they shift into Punitive Critic, then into Vulnerable Child (shame, fear), then use EDO behaviours to cope. In Bulimia, a person may start in EDO, flip into Detached Self-Soother following rejection, and return to EDO for compensation and control.

These sequences underscore the importance of viewing ED behaviours not simply as “maladaptive,” but as meaningful strategies rooted in developmental and emotional context [50].

### SMI-ED and the present study

To better assess these complex patterns in EDs, the SMI was adapted and expanded to include two ED-specific modes—EDO and Helpless Surrenderer—resulting in the 190-item SMI-ED [51]. This Australian adaptation improves diagnostic and treatment precision in ED. For use in clinical settings requiring brevity, a 64-item SMI-ED Short Form (SMI-ED-SF**)** was validated in Italy, retaining diagnostic utility while reducing respondent burden [52]. Vulnerable Child and coping mode scores on this measure significantly predict disordered eating behaviours [53].

Importantly, schema modes do not operate in isolation. Recent findings indicate that schema modes interact meaningfully with broader personality traits [26, 54, 55], influencing how individuals respond to stress and engage in treatment. This interaction warrants a more integrated approach to ED conceptualisation—one that incorporates both trait-based and schema-informed formulations.

### Current study

The present study evaluates the psychometric properties of the English version of the SMI-ED-SF (EN-SMI-ED-SF) in a community sample. Specifically:

- A Confirmatory Factor Analysis (CFA) was conducted to assess its factorial structure.
- A Multivariate Analysis of Variance (MANOVA) was performed to examine group differences across ED categories (No ED, AN, BN, BED, OSFED) on the sixteen EN-SMI-ED-SF dimensions.
- Finally, the relationship between schema modes and personality traits was explored to better understand the interplay between personality and mode-related functioning in ED presentations.

## Methods and materials

### Procedure

First, a back-to-back translation procedure was followed [56]. Second, a pilot test of clarity was conducted with 20 participants balanced by gender and age range. Participants completed the preliminary version of the EN-SMI-ED-SF online and provided structured feedback on item comprehensibility, wording clarity, and overall ease of understanding. No wording changes were deemed necessary based on this feedback. The final version is detailed in the supplementary materials (Supplementary 1). Third, participants were recruited via social media sites (e.g., Facebook, Twitter), online forums, and mailing lists, as well as through websites of various local clinical centers specialized in the treatment and rehabilitation of ED in the UK. Also, flyers were placed around University campuses and in clinical waiting rooms of local ED services – in line with previous studies [57, 58]. The recruitment was launched simultaneously across all channels. Participants were eligible if they: (A) were at least 18 years old; (B) spoke English as their native language; (C) completed the entire assessment battery without missing data; and (D) provided informed consent. After providing participants with a detailed description of the study and its inclusion and exclusion criteria, they were asked to acknowledge they had read the terms and conditions by signing an informed consent form. Following, subjects were asked to report demographic information and to answer the study questionnaires.

Participation was voluntary, and respondents were not compensated for participation. Informed consent was obtained from each study participant. This study was approved by the North of Scotland Research Ethics Committee (REC reference: 18/NS/0046; IRAS project ID: 241811).

### Sample size determination

The sample size was estimated a priori. Specifically, in the context of scale translation and validation, a commonly accepted guideline suggests that a sample size of approximately 500 participants may be adequate [59]. Furthermore, this sample size was also compared with the results of Monte Carlo simulations [60], which took into account the distribution of indicators, the estimator used, the number of items, the number of factors, and the number of items per factor. Considering model complexity, these simulations suggest that a minimum of 500 subjects is sufficient to accurately estimate the model parameters [59–61].

### Participants

The overall sample comprised 639 participants. The sample, included 71 males (11.1%) and 568 females (88.9%), aged between 18 and 79 years (*mean* = 32.08, *SD* = 11.04), with a Body Mass Index (BMI) ranging from 11.98 to 88.24 kg/m2 (*mean* = 24.39 kg/m^2^, *SD* = 8.41). More details are reported in Table 1.

**Table 1:** Sample’s descriptive statistics

	Descriptives (*N* = 639)	
Age (*M. SD*)	32.08	11.04
BMI (*M. SD*)	24.39	8.41
Civil status (*n* %)		
Single	332	52.0%
In a relationship/Married	275	43.0%
Separated	25	3.9%
Divorced	2	0.3%
Widowed	5	0.8%
Education (*n* %)		
Middle school	38	5.9%
High school	102	16.0%
Bachelor degree	482	75.4%
Master degree	13	2.0%
Ph.D.	4	0.6%
Occupation (*n* %)		
Student	204	31.9%
Dependent worker	251	39.3%
Entrepreneur	73	11.4%
Part-time worker	47	7.4%
Housewife	33	5.2%
Retired	10	1.6%
Unemployed	21	3.3%
Mental health professional Currently (*n* %)		
No	410	64.2%
Yes	229	35.8%
Mental health professional past year (*n* %)		
No	517	80.9%
Yes	122	19.1%
Eating disorder (*n* %)		
No ED	358	56.0%
Anorexia Nervosa (AN)	183	28.6%
Bulimia Nervosa (BN)	55	8.6%
Binge Eating Disorder (BED)	18	2.8%
Otherwise specified feeding and eating disorder (OSFED)	25	3.9%

### Measures

The following demographic information were collected: *age*, *gender*, *education*, *civil status*, and *employment status*. Moreover, participants were asked to self-report their *height* and *weight*—to calculate their BMI (Kg/m^2^)—as well as the *presence of a current diagnosis of an ED* [62].

### The schema mode inventory for eating disorders—short form (SMI-ED-SF)

The SMI-ED-SF [52] is a self-report questionnaire evaluating 16 schema modes clustered thematically according to the ST theoretical background across ED. It assesses (A) five innate child modes (1. Vulnerable Child—VC, 2. Angry Child—AC, 3. Enraged Child—EC, 4. Impulsive Child—IC, and 5. Undisciplined Child—UC); (B) two maladaptive (internalized/introject) modes (6. Punitive Mode PM and 7. Demanding Mode—DM); (C) seven maladaptive coping modes (8. Compliant Surrenderer—CS, 9. Helpless Surrenderer—HS, 10. Detached Protector—Det.P, 11. Detached Self-Soother—Det.SS, 12. Self-Aggrandizer—SA, 13. Bully and Attack—BA 14. Eating Disorder Overcontroller—EDO); and (D) two healthy factors (15. Happy Child—HC and 16. Healthy Adult—HA). Moreover, the SMI-ED-SF investigates two modes specifically created for individuals with ED (i.e., HS and EDO)—consisting exclusively of ED-specific statements [51]. Also, in line with the original version [51], each subscale comprises four general statements with 1 item representative of the ED population, except for those modes (IC and EC) only including either items retrieved from the original SMI [17]. Items are scored on a 6-point Likert scale ranging from 0 (“never or hardly ever”) to 5 (“all of the time”) and the higher the score, the more frequent the manifestations of the mode.

### The eating disorder diagnostic scale (EDDS)

The EDDS [63, 64] is a self-report questionnaire aimed at investigating AN, BN, and BED based on the DSM-IV criteria. The EDDS consists of a combination of 22 items with different response scales (e.g., Likert-type, yes/no, frequency scores, and open-ended questions like weight and height). The first four items assess the attitudinal symptoms of AN and BN in the past 3 months, such as fear of fatness and overvaluation of weight and shape. The next four items measure the frequency of uncontrollable consumption of a large amount of food, with a focus on the number of days per week over the past 6 months (BED and BN). The subsequent four items assess the frequency of behaviors that are used to compensate for binge eating over the past 3 months (BN), including vomiting, laxative or diuretic use, fasting, and excessive exercise. The EDDS consists of a diagnostic scale and a symptom composite scale and for the present study, only the latter was used. The symptom composite score indicates participants’ overall level of eating pathology with higher indicating greater eating pathology [63–65].

### The big five inventory (BFI)

The BFI [66] is one of the most used self-report questionnaires to assess personality traits. The BFI evaluates the Big Five personality traits—Neuroticism (N), Extraversion (E), Openness (O), Agreeableness (A), and Conscientiousness (C)—according to the five-factor model of personality theory. Its validity is supported by high correlations with other measures of the Big Five and peer ratings. The BFI is composed of 44 items and responses are captured on a 5-point Likert scale that spans from 1 (= “strongly disagree”) to 5 (= “strongly agree”). A high score on a subscale suggested that an individual exhibited strong characteristics of that particular personality dimension, whereas a low score indicated weaker traits in that area. Internal consistency values were satisfactory: N: McDonald’s omega = 0.872; E: McDonald’s omega = 0.799; O: McDonald’s omega = 0.786; A: McDonald’s omega = 0.755; C: McDonald’s omega = 0.740.

### The comprehensive assessment of acceptance and commitment therapy (CompACT)

The CompACT [67] is one of the most used questionnaires assessing psychological flexibility evaluating “triflex:” (1) openness to experience and detachment from literality (acceptance; defusion); (2) self-awareness and perspective-taking (present moment awareness; self-as-context); and (3) motivation and activation (values; committed action) [68, 69]. The CompACT is composed of 23 items and respondents rated their agreement for each item on a 7-point Likert-type scale from 0 (“strongly disagree”) to 6 (= “strongly agree”). According to the triflex, the CompACT contains three subscales: (1) openness to experience (OE), (2) behavioral awareness (BA), and (3) valued action (VA). Higher scores indicate greater psychological flexibility and its facets. Internal consistency values were satisfactory: OE: McDonald’s omega = 0.856; BA: McDonald’s omega = 0.864; VA: McDonald’s omega = 0.896.

### Statistical analysis

Statistical analyses were conducted using the R software environment. Based on the inclusion criteria, all observations were complete and without missing data.

A CFA was used to evaluate the factorial structure of the English version of the SMI-ED-SF, specifying a sixteen first-order correlated factors model [70]. The Robust Maximum Likelihood (MLM) estimator with Satorra-Bentler correction [71, 72] was used [70, 73, 74]. Model fit was assessed using traditional goodness-of-fit indices [74, 75], including the chi-square test (S-Bχ^2^), Root Mean Square Error of Approximation (RMSEA), Comparative Fit Index (CFI) and the Tucker-Lewis Index (TLI), and Standardized Root Mean Square Residual (SRMR), with the following recommended cutoff criteria: (A) non-significant S-Bχ^2^ (*p* <.50), (B) RMSEA ≤ 0.08, (C) CFI ≥ 0.90 (D) TLI ≥ 0.90, and (E) SRMR ≤ 0.08 [74, 76, 77]. Internal consistency was evaluated through McDonald’s omega [78], and item-total correlations (adjusted) were also calculated [79].

Moreover, a Multivariate Analysis of Variance (MANOVA) was performed to determine possible differences between ED conditions (No ED vs. AN vs. BN vs. BED vs. OSFED – independent variable) simultaneously on the sixteen EN-SMI-ED-SF scales (dependent variables). Wilks’ lambda (Λ) was chosen to test the multivariate effect [79, 80]. Moreover, focused contrasts with Games-Howell’s correction were performed [80, 81]. Partial eta-square (η^2^_p_) was used to quantify the difference in multiple comparisons—with the following benchmarks: small (η^2^_p_: 0.011 to 0.059), moderate (η^2^_p_: 0.060 to 0.139), and large (η^2^_p_ >0.140) [82].

Lastly, as an exploratory analysis, the association with personality traits was assessed using the Pearson correlation coefficient [79] and interpreted using Cohen’s benchmarks [82]: *r* <.10, trivial; *r* from 0.10 to 0.30, small; *r* from 0.30 to 0.50, moderate; *r* >.50; large.

## Results

### Structural validity

The EN-SMI-ED-SF model showed a good fit to the data. Despite the χ^2^ statistic resulted to be statistically significant [S-Bχ^2^ (1832) = 4491.647; *p* <.001], the other fit indices revealed a satisfactory fit to the data: the RMSEA = 0.048; 90%CI 0.046–0.049; *p*(RMSEA < 0.05) = 0.992 *ns*, the CFI = 0.903, the TLI = 0.894, and the SRMR = 0.066. All the items’ standardized factor loadings were statistically significantly associated with their latent variable and ranged from 0.613 (item#50; SO) to 0.947 (item#63; EDO) – Table 2. Also, the degree of explained variance ranged from ranged from 0.375 (item#50; SO) to 0.897 (item#63; EDO).

**Table 2:** Item descriptive statistics, psychometric properties, and CFA results

	Descriptives				Properties	CFA	
	M	SD	SK	K	*r*(it-tot)	λ	*R* ^2^
*VC—vulnerable child*							
Item#1	2.39	1.31	0.08	− 0.65	0.751	0.817	0.668
Item#2	2.02	1.83	0.35	− 1.28	0.641	0.714	0.510
Item#3	2.23	1.54	0.23	− 0.94	0.814	0.889	0.790
Item#4	2.04	1.51	0.36	− 0.82	0.792	0.862	0.744
*AC—angry child*							
Item#5	1.37	1.54	0.96	− 0.16	0.631	0.753	0.567
Item#6	1.57	1.40	0.71	− 0.32	0.700	0.741	0.549
Item#7	1.58	1.51	0.73	− 0.43	0.800	0.885	0.783
Item#8	0.81	1.20	1.71	2.59	0.665	0.724	0.524
*EC—enraged child*							
Item#9	0.49	0.92	2.21	5.01	0.686	0.740	0.548
Item#10	0.70	1.03	1.65	2.55	0.845	0.916	0.840
Item#11	0.59	0.97	1.98	4.06	0.823	0.897	0.804
Item#12	1.01	1.13	1.15	0.96	0.680	0.737	0.543
*IC—impulsive child*							
Item#13	1.23	1.23	1.00	0.53	0.762	0.816	0.665
Item#14	1.24	1.35	1.06	0.43	0.741	0.811	0.657
Item#15	1.21	1.23	1.17	1.12	0.817	0.876	0.768
Item#16	1.32	1.25	0.96	0.56	0.764	0.813	0.661
*UC—undisciplined child*							
Item#17	1.50	1.44	0.68	− 0.43	0.633	0.722	0.521
Item#18	1.75	1.39	0.52	− 0.58	0.722	0.820	0.673
Item#19	1.03	1.17	1.11	0.73	0.588	0.667	0.445
Item#20	1.85	1.48	0.59	−0.57	0.609	0.706	0.498
*HC—happy child*							
Item#21	2.82	1.42	− 0.21	− 0.93	0.780	0.826	0.683
Item#22	2.72	1.47	− 0.23	− 0.92	0.670	0.715	0.512
Item#23	2.21	1.39	0.14	− 0.90	0.836	0.899	0.809
Item#24	2.57	1.40	− 0.05	− 0.91	0.749	0.838	0.702
*PM—punitive mode*							
Item#25	1.67	1.73	0.70	− 0.83	0.852	0.881	0.777
Item#26	1.67	1.70	0.71	− 0.78	0.836	0.865	0.748
Item#27	1.22	1.55	1.05	− 0.16	0.905	0.937	0.878
Item#28	1.44	1.68	0.80	− 0.75	0.893	0.938	0.880
*DM—demanding mode*							
Item#29	2.38	1.79	0.11	− 1.35	0.713	0.825	0.680
Item#30	2.19	1.77	0.24	− 1.27	0.761	0.890	0.792
Item#31	2.73	1.53	−0.12	− 1.01	0.676	0.672	0.452
Item#32	3.02	1.57	−0.37	− 0.96	0.662	0.683	0.467
*HA—healthy adult*							
Item#33	2.92	1.38	− 0.39	− 0.77	0.765	0.839	0.703
Item#34	2.49	1.20	− 0.07	− 0.73	0.647	0.689	0.474
Item#35	2.66	1.43	− 0.20	− 0.92	0.796	0.861	0.742
Item#36	2.94	1.34	− 0.23	− 0.67	0.762	0.831	0.690
*CS—compliant surrender*							
Item#37	2.79	1.22	0.07	− 0.57	0.681	0.694	0.482
Item#38	2.48	1.34	0.13	− 0.72	0.700	0.728	0.530
Item#39	3.33	1.38	− 0.45	− 0.73	0.685	0.744	0.553
Item#40	2.44	1.77	0.00	− 1.35	0.666	0.840	0.706
*Det.P—detached protector*							
Item#41	2.56	1.51	− 0.02	− 0.92	0.804	0.873	0.762
Item#42	2.23	1.56	0.20	− 0.95	0.706	0.744	0.554
Item#43	1.80	1.62	0.55	− 0.85	0.810	0.871	0.758
Item#44	1.16	1.38	1.08	0.31	0.703	0.778	0.605
*Det.SS—detached self-soother*							
Item#45	2.20	1.88	0.21	− 1.43	0.681	0.816	0.666
Item#46	2.56	1.63	− 0.04	− 1.15	0.647	0.651	0.424
Item#47	1.88	1.71	0.47	− 1.07	0.657	0.729	0.532
Item#48	2.75	1.65	− 0.15	− 1.19	0.756	0.831	0.690
*SA—self-aggrandizer*							
Item#49	2.16	1.33	0.31	− 0.57	0.566	0.641	0.411
Item#50	0.95	1.35	1.52	1.54	0.500	0.613	0.375
Item#51	1.92	1.86	0.43	− 1.31	0.494	0.666	0.443
Item#52	1.57	1.32	0.68	− 0.15	0.580	0.685	0.470
*BA—bully and attack*							
Item#53	0.51	1.00	2.60	7.35	0.732	0.820	0.673
Item#54	0.49	0.88	2.36	6.62	0.640	0.707	0.499
Item#55	0.67	1.11	2.09	4.41	0.792	0.862	0.743
Item#56	0.93	1.27	1.50	1.68	0.668	0.749	0.560
*HS—helpless surrenderer*							
Item#57	2.30	1.65	0.19	− 1.12	0.664	0.711	0.506
Item#58	2.14	1.46	0.33	− 0.75	0.644	0.675	0.456
Item#59	2.23	1.60	0.27	− 1.04	0.566	0.714	0.510
Item#60	1.95	1.67	0.45	− 1.02	0.716	0.828	0.686
*EDO—eating disorder overcontroller*							
Item#61	1.86	1.87	0.50	− 1.25	0.867	0.899	0.808
Item#62	2.12	1.90	0.29	− 1.40	0.903	0.939	0.882
Item#63	2.07	1.87	0.31	− 1.36	0.914	0.947	0.897
Item#64	1.64	1.82	0.68	− 1.00	0.820	0.846	0.716

M = mean; SD = standard deviation; SK = Skewness, K = Kurtosis; *r*(it-tot) = adjusted item-total correlation; λ = standardized factor loading; *R*^2^ = item explained variance

Moreover, the correlation analysis showed small-to-large associations among the sixteen EN-SMI-ED-SF dimensions—see Table 3. Also, reliability analysis revealed satisfying results for all of the EN-SMI-ED-SF scales. Indeed, the McDonald’s omega ranges from 0.729 (SA scale) to 0.950 (EDO scale)—Table 4.

**Table 3:** Correlation analysis between SMI-SF dimensions and internal consistency (McDonald’s omega)

		1	2	3	4	5	6	7	8	9	10	11	12	13	14	15	16	ω
1	VC	–																0.881
2	AC	0.621	–															0.861
3	EC	0.359	0.599	–														0.892
4	IC	0.455	0.509	0.565	–													0.896
5	UC	0.445	0.404	0.385	0.597	–												0.820
6	HC	− 0.736	− 0.511	− 0.331	− 0.365	− 0.401	–											0.892
7	PM	0.720	0.459	0.262	0.347	0.233	− 0.555	–										0.945
8	DM	0.660	0.424	0.239	0.309	0.196	− 0.506	0.960	–									0.862
9	HA	− 0.703	− 0.469	− 0.311	− 0.347	− 0.384	0.785	− 0.606	− 0.535	–								0.886
10	CS	0.603	0.363	0.166	0.240	0.338	− 0.501	0.592	0.563	− 0.582	–							0.842
11	Det.P	0.763	0.555	0.352	0.444	0.451	− 0.739	0.611	0.556	− 0.682	0.563	–						0.892
12	Det.SS	0.746	0.546	0.338	0.409	0.386	− 0.622	0.697	0.673	− 0.622	0.562	0.674	–					0.848
13	SA	0.427	0.465	0.404	0.441	0.307	− 0.343	0.507	0.506	− 0.360	0.248	0.459	0.458	–				0.729
14	BA	0.311	0.442	0.381	0.412	0.299	− 0.246	0.272	0.257	− 0.202	0.104^*^	0.385	0.286	0.647	–			0.863
15	HS	0.656	0.532	0.419	0.516	0.503	− 0.550	0.575	0.554	− 0.547	0.473	0.616	0.608	0.566	0.399	–		0.827
16	EDO	0.698	0.450	0.236	0.366	0.316	− 0.514	0.740	0.690	− 0.548	0.516	0.594	0.679	0.506	0.314	0.608	–	0.950

All correlations are significant at *p* <.001, except for *(*p* <.010). ω = McDonald’s Omega. VC = Vulnerable child; AC = angry child; EC = enraged child; IC = impulsive child; UC = undisciplined child; HC = happy child; PM = punitive mode; DM = demanding mode; HA = healthy adult; CS = compliant surrender; Det.P = detached protector; Det.SS = detached self-soother; SA = self-aggrandizer; BA = bully and attack; HS = helpless surrenderer; EDO = eating disorder overcontroller

**Table 4:** MANOVA results

	NO ED	AN	BN	BED	OSFED	MANOVA		
	M(SD)	M(SD)	M(SD)	M(SD)	M(SD)	F	η^2^_p_	Contrasts ^a^
VC	1.53(1.13)	3.04(1.09)	2.93(0.97)	3.07(1.42)	2.65(1.30)	67.323^***^	0.298	No ED < AN^***^; NO ED < BN^***^; No ED < BED^**^; NO ED < OSFED^**^;
AC	1.01(1.05)	1.64(1.19)	1.85(1.25)	2.42(1.28)	1.65(1.21)	18.306^***^	0.104	NO ED < AN^***^; NO ED < BN^***^; NO ED < BED^**^;
EC	0.60(0.78)	0.77(0.98)	0.84(0.92)	1.43(1.25)	0.75(0.70)	5.028^**^	0.031	
IC	1.05(0.99)	1.41(1.18)	1.75(1.28)	2.50(1.08)	0.99(0.68)	13.826^***^	0.080	NO ED < AN^**^; NO ED < BN^**^; NO ED < BED^***^; AN < BED^**^; BN > OSFED^**^; BED > OSFED^**^;
UC	1.40(1.07)	1.58(1.05)	1.85(1.22)	2.78(1.04)	1.56(1.06)	8.603^***^	0.051	NO ED < BED^***^; AN < BED^**^; BN < BED^*^; BED > OSFED^**^;
HC	3.04(1.15)	1.99(1.04)	1.95(0.98)	2.04(1.14)	2.00(1.46)	35.127^***^	0.181	NO ED > AN^***^; NO ED > BN^***^; NO ED > BED^**^; NO ED > OSFED^**^;
PM	1.50(1.09)	3.22(1.25)	2.91(1.19)	2.13(1.23)	2.93(1.50)	74.628^***^	0.320	NO ED < AN^***^; NO ED < BN^***^; NO ED < OSFED^**^; AN > BED^*^;
DM	1.95(1.18)	3.51(1.21)	3.32(1.14)	2.58(1.21)	3.21(1.41)	59.717^***^	0.274	NO ED < AN^***^; NO ED < BN^***^; NO ED < OSFED^**^; AN > BED^*^;
HA	3.20(1.02)	2.11(1.02)	2.35(1.03)	2.39(1.12)	2.22(1.20)	38.945^***^	0.197	NO ED > AN^***^; NO ED > BN^***^; NO ED > OSFED^**^;
CS	2.36(1.08)	3.33(1.07)	3.14(0.99)	2.85(1.40)	3.52(1.26)	29.501^***^	0.157	NO ED < AN^***^; NO ED < BN^***^; NO ED < OSFED^**^;
Det.P	1.47(1.21)	2.59(1.15)	2.50(1.15)	2.51(1.36)	2.30(1.41)	31.912^***^	0.168	NO ED < AN^***^; NO ED < BN^***^; NO ED < BED^**^;
Det.SS	1.70(1.24)	3.15(1.17)	3.46(1.09)	3.14(1.11)	2.77(1.52)	59.963^***^	0.274	NO ED < AN^***^; NO ED < BN^***^; NO ED < BED^***^; NO ED < OSFED^**^;
SA	1.41(1.01)	1.99(1.20)	1.91(1.01)	2.03(1.01)	1.70(1.05)	10.364^***^	0.061	NO ED < AN^***^; NO ED < BN^**^;
BA	0.59(0.87)	0.75(0.97)	0.77(0.80)	0.68(1.17)	0.45(0.49)	1.529	0.010	
HS	1.73(1.22)	2.69(1.16)	2.73(1.12)	3.04(1.05)	2.42(1.30)	26.488^***^	0.143	NO ED < AN^***^; NO ED < BN^***^; NO ED < BED^**^;
EDO	1.03(1.38)	3.17(1.44)	3.02(1.33)	2.89(1.89)	2.41(1.33)	82.706^***^	0.343	NO ED < AN^***^; NO ED < BN***; NO ED < BED^**^; NO ED < OSFED^***^;

***p <.001; **p <.010; *p <.050; Contrasts ^a^ = Games-Howell post-hoc analysis. *M* = mean; *SD* = Standard deviation; NO ED = No eating disorder; AN = Anorexia nervosa; BN = Bulimia Nervosa; BED = Binge eating disorder; OSFED = Otherwise specified feeding and eating disorder. VC = Vulnerable child; AC = angry child; EC = enraged child; IC = impulsive child; UC = undisciplined child; HC = happy child; PM = punitive mode; DM = demanding mode; HA = healthy adult; CS = compliant surrender; Det.P = detached protector; Det.SS = detached self-soother; SA = self-aggrandizer; BA = bully and attack; HS = helpless surrenderer; EDO = eating disorder overcontroller

Lastly, large associations were found between the EDDS symptoms composite score and the EDO scale (*r* =.633; *p* <.001), the PM scale (*r* =.588; *p* <.001), the Det.SS scale (*r* =.570; *p* <.001), the VC scale (*r* =.563; *p* <.001), and the DM scale (*r* =.531; *p* <.001). Also, all of the EN-SMI-ED-SF scales revealed a statistically significant linear association with the EDDS symptoms composite score.

### MANOVA results

**Fig. 1:**
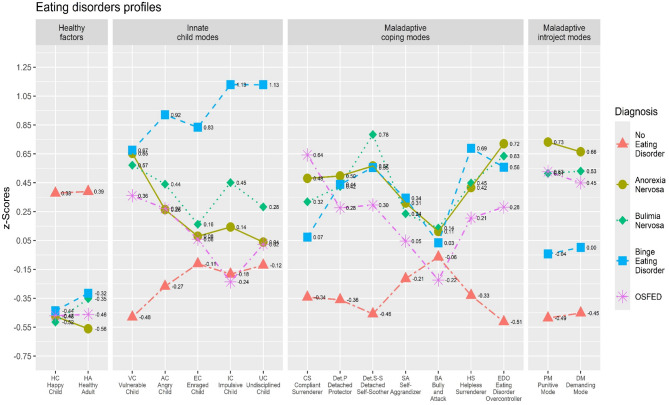
Trends of the mean scores (z-scores) for the sixteen EN-SMI-ED-SF dimensions across the five groups of subjects with an ED diagnosis

The raw score of each variable was almost normally distributed and their relationships were substantially linear. The Box’s *M* was statistically significant (*M* = 940.908, *F* = 1.375, *p* <.001) – however, it should be noted that MANOVA is robust to violations of assumptions [79, 80]. Thus, considering these results, the MANOVA was performed. A statistically significant multivariate effect was found: Λ = 0.478, *F* = 7.886, *p* <.001; η^2^_p_ = 0.169 (large effect size) with a statistically significant between groups difference for the VC scale [*F* = 67.323, *p* <.001; η^2^_p_ = 0.298 (large effect size)]; the AC scale [*F* = 18.306, *p* <.001; η^2^_p_ = 0.104 (moderate effect size)], the EC scale [*F* = 5.028, *p* =.001; η^2^_p_ = 0.031 (small effect size)], the IC scale [*F* = 13.826, *p* <.001; η^2^_p_ = 0.080 (moderate effect size)], the UC scale [*F* = 8.603, *p* <.001; η^2^_p_ = 0.051 (small effect size)], the HC scale [*F* = 35.127, *p* <.001; η^2^_p_ = 0.181 (large effect size)], the PM scale [*F* = 74.628, *p* <.001; η^2^_p_ = 0.320 (large effect size)], the DM scale [*F* = 59.717, *p* <.001; η^2^_p_ = 0.274 (large effect size)], the HA scale [*F* = 38.945, *p* <.001; η^2^_p_ = 0.197 (large effect size)], the CS scale [*F* = 29.501, *p* <.001; η^2^_p_ = 0.157 (large effect size)], the Det.P scale [*F* = 31.912, *p* <.001; η^2^_p_ = 0.168 (large effect size)], the Det.SS scale [*F* = 59.963, *p* <.001; η^2^_p_ = 0.274 (large effect size)], the SA scale [*F* = 10.364, *p* <.001; η^2^_p_ = 0.061 (moderate effect size)], the BA scale [*F* = 1.529, *p* =.192; η^2^_p_ = 0.010 (trivial effect size)], the HS scale [*F* = 26.488, *p* <.001; η^2^_p_ = 0.143 (large effect size)], and the EDO scale [*F* = 82.706, *p* <.001; η^2^_p_ = 0.343 (large effect size)]. Detailed results are reported in Table 4; Fig. 1.

### Associations with personality traits

Regarding the association between the sixteen EN-SMI-ED-SF scales and personality traits, small-to-large correlations were observed.

In particular, considering the association between the EN-SMI-ED-SF scales and the BFI-N scale, the strongest correlations were found with the VC scale (*r* =.679; *p* <.001), the HA scale (*r* = −.670; *p* <.001), and the HC scale (*r* = −.653; *p* <.001). Moreover, considering the association between the EN-SMI-ED-SF scales and the BFI-O scale, the strongest correlations were found with the HA scale (*r* =.298; *p* <.001) and the HC scale (*r* =.222; *p* <.001). Also, considering the association between the EN-SMI-ED-SF scales and the BFI-E scale, the strongest correlations were found with the Det.P scale (*r* = −.535; *p* <.001), the HA scale (*r* =.513; *p* <.001), and the HC scale (*r* =.475; *p* <.001).

Considering the association between the EN-SMI-ED-SF scales and the BFI-A scale, the strongest correlations were found with the SA scale (*r* = −.527; *p* <.001), the BA scale (*r* = −.470; *p* <.001), and the EC scale (*r* = −.376; *p* <.001). Furthermore, considering the association between the EN-SMI-ED-SF scales and the BFI-C scale, the strongest correlations were found with the UC scale (*r* = −.557; *p* <.001) and the IC scale (*r* = −.376; *p* <.001). Lastly, considering the association between the EN-SMI-ED-SF scales and the CompACT total score scale, the strongest correlations were found with the HA scale (*r* =.769; *p* <.001), the VC scale (*r* = −.762; *p* <.001), and HC scale (*r* =.752; *p* <.001). Detailed results are reported in Table 5.

**Table 5:** Correlation analysis between EN-SMI-ED-SF dimensions and personality traits

	EDDS	BFI - *N*	BFI - O	BFI - E	BFI - A	BFI - C	CompACT Total	CompACT - OE	CompACT - BA	CompACT - VA
VC	0.563	0.679	− 0.138	− 0.432	− 0.213	− 0.211	− 0.762	− 0.742	− 0.576	− 0.574
AC	0.359	0.506	− 0.011^§^	− 0.257	− 0.333	− 0.215	− 0.562	− 0.539	− 0.470	− 0.401
EC	0.187	0.400	− 0.048^§^	− 0.154	− 0.376	− 0.216	− 0.358	− 0.342	− 0.305	− 0.255
IC	0.297	0.380	− 0.101^**^	− 0.086^*^	− 0.359	− 0.378	− 0.481	− 0.402	− 0.417	− 0.421
UC	0.226	0.363	− 0.148	− 0.178	− 0.162	− 0.557	− 0.499	− 0.382	− 0.448	− 0.479
HC	− 0.422	− 0.653	0.222	0.475	0.277	0.249	0.752	0.698	0.551	0.631
PM	0.588	0.556	− 0.124^**^	− 0.305	− 0.172	0.028	− 0.626	− 0.649	− 0.480	− 0.407
DM	0.531	0.538	− 0.112^**^	− 0.262	− 0.166	0.074	− 0.586	− 0.631	− 0.447	− 0.350
HA	− 0.462	− 0.670	0.298	0.513	0.266	0.261	0.769	0.697	0.552	0.679
CS	0.423	0.512	− 0.142	− 0.416	0.064^§^	− 0.192	− 0.616	− 0.624	− 0.444	− 0.445
Det.P	0.488	0.577	− 0.167	− 0.535	− 0.324	− 0.236	− 0.739	− 0.676	− 0.585	− 0.602
Det.ss	0.570	0.584	− 0.131^**^	− 0.313	− 0.171	− 0.158	− 0.707	− 0.725	− 0.527	− 0.484
SA	0.340	0.398	− 0.068^§^	− 0.142	− 0.527	− 0.168	− 0.448	− 0.421	− 0.381	− 0.329
BA	0.247	0.222	0.018^§^	− 0.061^§^	− 0.470	− 0.159	− 0.312	− 0.267	− 0.311	− 0.233
HS	0.397	0.611	− 0.152	− 0.330	− 0.279	− 0.295	− 0.672	− 0.652	− 0.504	− 0.514
EDO	0.633	0.467	− 0.119^**^	− 0.249	− 0.143	− 0.121	− 0.618	− 0.612	− 0.470	− 0.448

All correlations are significant at *p* <.001, except for: **p <.010 and *p <.050; ^§^*p* <.050. VC = Vulnerable child; AC = angry child; EC = enraged child; IC = impulsive child; UC = undisciplined child; HC = happy child; PM = punitive mode; DM = demanding mode; HA = healthy adult; CS = compliant surrender; Det.P = detached protector; Det.SS = detached self-soother; SA = self-aggrandizer; BA = bully and attack; HS = helpless surrenderer; EDO = eating disorder overcontroller. EDDS = EDDS symptoms composite score; BFI – N = BFI Neuroticism scale; BFI – E = BFI Extraversion scale; BFI – O = BFI Openness scale; BFI – A = BFI Agreeableness scale; BFI – C = BFI Conscientiousness scale; CompACT Total = CompACT Total score; CompACT – OE = CompACT openness to experience scale; CompACT – BA = CompACT behavioral awareness scale; CompACT – VA = CompACT valued action scale

## Discussion

ST is increasingly establishing itself as an effective psychotherapeutic approach for treating various psychological (and psychopathological) conditions, such as anxiety, depression, PDs, and ED [83–85]. ST-informed literature emphasizes the interplay between ED and PD features, highlighting how maladaptive schema modes and coping styles may reinforce one another and contribute to the persistence of disordered eating behaviors [26, 86]. In this context, only one specific self-report instrument based on ST has been developed to assess schema modes in ED: the SMI-ED [51] and its short-form version (SMI-ED-SF) [52]. Therefore, the present study aimed to evaluate the factor structure and psychometric properties of the English version of the SMI-ED-SF. Additionally, the study aimed to examine the associations between schema modes, different eating patterns conditions, and personality traits.

### Factorial structure of the EN-SMI-ED-SF

The results suggest that the test of the factorial structure of the English version of the SMI-ED-SF showed satisfying fit indices. The EN-SMI-ED-SF demonstrated a stable and conceptually coherent factorial structure with sixteen interrelated first-order factors, closely mirroring the factorial results obtained for its longer English version [51] and its corresponding Italian short-form version [52]. Notably, this factorial structure is replicated both across different forms (with a varying number of items) and across different cultures (Italian vs. English), supporting the potential for cross-cultural studies—although these would first require testing for invariance.

Although the fit indices are satisfactory and, for the most part, in line with the original Italian version, the English version of the SMI-ED-SF shows a higher CFI value than its Italian counterpart—albeit only slightly above the conventional acceptability threshold (CFI = 0.903)—and the TLI was slightly under the suggested threshold (TLI = 0.894). This can be explained by the fact that, as suggested by Kenny and McCoach (2003), the number of items/indicators (consequently, the number of parameters) within a CFA model appears to negatively affect their performance, yielding lower-than-usual values [87–89]. Also, a slightly lower TLI than CFI is quite common and reflects that the TLI evaluates model parsimony in addition to its overall goodness of fit. Both values around 0.90 indicate an acceptable fit, but the slightly lower TLI signals a stricter control of model complexity.

The adequacy of fit is further supported by others goodness of fit indices (RMSEA and SRMR) as well as strong standardized factor loadings, particularly for high-risk schema modes in ED, which were all statistically significant, indicating that the items contribute meaningfully to their respective latent variables.

Interestingly, higher items mean scores are observed in adaptive schema modes (HC, HA) and lower endorsement of maladaptive aggressive modes (EC, BA), suggesting that participants engage more frequently in self-regulated and socially attuned schema responses. This variability in schema mode endorsement suggests that the EN-SMI-ED-SF is effective in capturing distinct, less adaptive cognitive-affective patterns, while also highlighting potential therapeutic strengths. The EDO and PM scales appear to be the most psychometrically robust, aligning with the literature on maladaptive perfectionism and punitive self-evaluation in ED [90, 91]. Lastly, the analysis of internal consistency, conducted using McDonald’s omega coefficient, showed that all scales of the EN-SMI-ED-SF exhibit excellent levels of coherence.

### Interconnected schema patterns: risk and protective dimensions

The correlation analysis between EN-SMI-ED-SF dimensions provides evidence of the model’s validity and reliability. The results demonstrate consistent relationships between schema modes, supporting their theoretical interconnectedness while also revealing key distinctions between adaptive and maladaptive modes. Indeed, strong positive associations among maladaptive schema modes were observed, while adaptive schema modes show inverse relationships with dysfunctional patterns. The highest correlations were found between PM and DM, indicating that self-criticism and high personal demands strongly co-exist. The EDO scale was also highly correlated with PM and DM, reinforcing its link to perfectionism and self-directed criticism in ED.

Moreover, strong associations were detected between Det.SS and PM, suggesting that avoidance strategies often co-exist with punitive self-perceptions. The robust inverse associations between adaptive (HC and HA) and maladaptive schema modes (EDO, PM, Det.P, and VC) indicated that higher levels of psychological well-being are associated with lower endorsement of maladaptive schema modes. This highlights the potential of positive self-schemas as protective resources, suggesting that enhancing them may reduce reliance on less adaptive coping patterns [92].

### Group differences in schema modes among subjects with ED

Across all ED groups, higher scores in maladaptive schema modes (e.g., VC) were observed compared to non-ED individuals. Particularly, the significantly high EDO scores in AN and BN suggest that excessive control over eating and emotions is a core feature of restrictive and purging behaviors. The Det.P and Det.SS modes were also significantly elevated in ED groups, with Det.SS showing one of the highest effect sizes in BN and BED. These findings support the avoidance model of ED, suggesting that emotional detachment strategies (e.g., binge eating, food restriction) may be used to regulate distressing emotions.

Conversely, adaptive schema modes (HA and HC) were lower in ED groups, with the largest differences observed between non-ED individuals and AN/BN groups. These findings reinforce the importance of fostering emotional resilience and self-compassion through the development of adaptive schema modes as part of a strengths-based treatment approach.

Individuals diagnosed with AN exhibited the highest scores in the PM and DM, consistent with their association with perfectionism and self-punishment. The elevated PM suggests that individuals with AN engage in harsh self-criticism and rigid self-evaluation, while the DM reflects high personal expectations and an excessive focus on achievement and control, particularly over body weight and food intake. These cognitive patterns align with previous research linking perfectionistic tendencies to the development and maintenance of AN [93–95].

Individuals diagnosed with BED exhibited the highest scores on the IC and UC modes. These findings indicate that difficulty in emotional regulation and poor impulse control are key characteristics of binge eating behaviors. The IC mode reflects acting on immediate urges without considering long-term consequences. In contrast, the UC mode suggests a lack of self-control and difficulty adhering to structured behaviors, which may contribute to episodes of binge eating. These results are consistent with models of BED that emphasize uncontrolled responses to emotional dysregulation as a central maintaining factor [96–98].

Both BN and BED groups displayed higher scores in the Det.SS mode, indicating a greater tendency toward emotional avoidance through less adaptive behaviors. The Det.SS mode represents a coping strategy in which individuals attempt to suppress or escape distressing emotions by engaging in external soothing behaviors, such as binge eating or purging. The elevated scores in BN and BED groups suggest that binge eating episodes may function as an avoidant response to psychological distress, reinforcing previous findings that link emotional avoidance and dissociation with disordered eating behaviors [99, 100].

### Association between schema modes and personality traits

The correlation analysis between SMI-SF dimensions and personality traits provides important insights into the relationship between schema modes, eating disorder symptoms (EDDS), personality traits (BFI), and psychological flexibility (CompACT scales).

Maladaptive schema modes (VC, PM, and Det.P) showed the strongest positive correlations with neuroticism, suggesting that individuals with high emotional instability are more likely to experience self-critical and avoidant schema modes. Conversely, adaptive schema modes (HA and HC) were negatively correlated with neuroticism, indicating that emotionally stable individuals exhibit stronger adaptive self-schemas. Neuroticism, and in particular negative affectivity, has been highlighted in previous studies as a vulnerability factor in the development of ED [101–104]. Elevated levels of this trait in individuals with ED have been linked to the interaction of genetic and early environmental factors, including unmet childhood needs associated with care/nurturance, safety/protection, and emotional expression [105, 106]. In contrast, BFI Extraversion (BFI-E) and BFI Agreeableness (BFI-A) were negatively correlated with maladaptive schema modes, reinforcing their role as protective interpersonal traits. BFI-E was most strongly negatively associated with Det.P, CS, and VC, suggesting that individuals with low extraversion are more likely to cope through emotional detachment and social withdrawal. BFI-A showed significant negative associations with PM, DM, and Det.P, reinforcing the link between low agreeableness and punitive and perfectionistic self-criticism and detached coping.

These findings support the role of neuroticism as a key vulnerability factor associated with the activation of maladaptive schema modes, and interventions that target these modes may help reduce emotional dysregulation and reinforce psychological stability. Extraversion and agreeableness would, instead, represent protective traits associated with greater emotional resilience. Indeed, the central focus in schema therapy is to facilitate a strong reparenting ‘bond’ between the individual’s HA and VC, which in turn facilitates connectedness, flexibility, and capacity for adaptive emotional expression [107].

The CompACT total score and subscales exhibited strong negative correlations with maladaptive schema modes. Particularly, the CompACT-OE and CompACT-BA subscales were strongly negatively correlated with VC, PM, and Det.P, suggesting that greater psychological flexibility is associated with reduced self-critical and avoidant schema modes. The EDO mode was negatively correlated with CompACT total, CompACT-BA, and CompACT-VA, indicating that greater flexibility in valued actions is associated with lower ED overcontrol tendencies. This highlights the role of coping modes in hindering self-reflection, whereby feeling thinking and actions tend to follow rigid, avoidant patterns designed to disconnect schema activation [107]. In contrast, HA and HC modes showed strong positive correlations with CompACT total score, reinforcing the idea that higher psychological flexibility is linked to more adaptive self-schemas. Fostering psychological flexibility may, therefore, promote healthier self-schemas and reduce reliance on avoidance-based coping strategies. Indeed, the development of capacity for self-reflection and mentalization have been highlighted as fundamental aspects in ST through strengthening the HA mode [108].

Lastly, it should be noted that unlike the primary analyses (i.e., CFA and MANOVA), which were based on previous literature, these correlation analyses were exploratory in nature. For this reason, no multiple-comparison correction was applied. This decision reflects both the conceptual interrelatedness of the psychological constructs examined and the exploratory purpose of the analyses. Because the measures are theoretically and statistically related, strict corrections (e.g., Bonferroni) may not be appropriate, as they assume independence across tests and can substantially reduce power, potentially masking meaningful patterns that warrant further investigation.

### Limitations and strengths

The reliance on self-report measures additionally precluded formal diagnostic assessments and comprehensive symptom evaluations. Additionally, social desirability bias could not be ruled out. Furthermore, the naturalistic and online recruitment method limited the ability to control for gender distribution which resulted in a sample with a particularly pronounced female prevalence (88.9%); indeed, men represented a small proportion of the sample. While this mirrors the gender distribution typically observed in clinical ED populations [109–111], it nonetheless restricts the generalizability of the findings to more gender-diverse or male-identifying groups. To enhance the instrument’s applicability and inclusivity, future research should assess measurement invariance across gender identities. Another limitation lies in the recruitment strategy: the survey was intentionally designed to include participants who self-identified as experiencing disordered eating, including subthreshold cases. While this approach enhanced ecological validity and allowed for the inclusion of individuals often excluded from clinical studies, it limits the generalizability of findings to strictly defined clinical populations. Another limitation concerns the assessment of ED diagnoses, which were self-reported by participants in the sociodemographic form and were not independently verified. Although this approach may reduce diagnostic accuracy, it reflects procedures commonly adopted in previous research and is consistent with established guidelines for online data collection. Furthermore, the use of online recruitment may have introduced a degree of self-selection bias. Individuals in the pre-contemplative stage—those with limited insight or awareness of disordered eating—may have been under-represented [112–115]. It is also plausible that individuals with more severe ED presentations who, due to social withdrawal or heightened distress, might be less likely to engage in online research. The relatively low representation of participants reporting binge eating behaviors may also have reduced the statistical power to detect differences within this subgroup, possibly reflecting the greater shame or secrecy that can characterize this symptomatology and reduce participation willingness.

The cross-sectional design limits conclusions regarding temporal stability, clinical sensitivity, and predictive validity of schema modes. Although the measure can inform case conceptualization and support personalized treatment planning, caution is warranted when interpreting these results in a predictive context. Future longitudinal studies are needed to assess the sensitivity of schema modes to therapeutic change and their role in the evolution of ED symptomatology.

To further strengthen the clinical utility of the EN-SMI-ED-SF, future studies should include structured diagnostic interviews to ensure adequate representation across ED diagnostic subtypes. This would allow exploration of whether distinct schema mode patterns emerge across diagnostic categories. Nevertheless, in light of increasing interest in transdiagnostic models of EDs, such distinctions may remain blurred [116]. In this regard, the tool’s capacity to capture shared mechanisms across ED types can be seen as a strength rather than a limitation. Although some degree of overlap between schema mode items is theoretically expected, future studies should further clarify the internal structure of the tool and examine its discriminant validity [117, 118]. While convergent validity is supported, the lack of discriminant validity testing in this study represents an additional area for future investigation to enhance the instrument’s overall construct validity.

Lastly, a significant limitation concerns the lack of the evaluation of measurement invariance among groups: indeed, formal equivalence of factor loadings and item intercepts (metric and scalar invariance) was not tested through constrained multi-group models. This limitation is particularly relevant for subgroups with reduced sample sizes (BED *n* = 18, OSFED *n* = 25), which preclude statistically robust multi-group invariance testing. When strong invariance assumptions are violated, inaccurate inferences may occur. Consequently, MANOVA results should be cautiously interpreted considering this methodological limitation, as observed differences may reflect both genuine clinical variations and potential uncontrolled measurement bias. Future research should include formal measurement invariance tests with adequately powered samples for all diagnostic subgroups.

Among the key strengths of this study is that it provides the first English-language validation of the SMI-ED-SF—a self-report tool grounded in schema therapy and tailored specifically to assess schema modes relevant to disordered eating, while maintaining a transdiagnostic perspective. The results closely replicate the findings from the Italian validation [52], reinforcing its psychometric robustness and applicability across different samples. The strong model fit and internal consistency of the EN-SMI-ED-SF support its structural validity and reliability in assessing both adaptive and maladaptive modes in individuals with disordered eating. The tool offers potential clinical and economic benefits by supporting early identification of critical schema patterns in ED presentations.

If routinely integrated into assessment batteries, the EN-SMI-ED-SF could assist clinicians in developing more precise, mode-informed interventions. Additional strengths of this research include the large sample size, which allowed for accurate statistical model estimates, the strong psychometric properties of the instrument, and the use of rigorous and well-established statistical methods in line with current guidelines. Moreover, the observed associations with personality traits and psychological flexibility further underscore the tool’s clinical relevance, offering new avenues for tailoring interventions based on individual schema mode profiles and underlying personality dimensions.

### Clinical implications

These findings reinforce the utility of the SMI-ED-SF as a reliable and valid instrument for assessing schema modes associated with disordered eating behaviours [51, 52]. The accurate identification of schema modes enables clinicians to develop personalized case conceptualizations, fostering a deeper understanding of how eating behaviours relate to different parts of the self. This facilitates recognition of the multiple psychological functions that disordered eating can serve—often as strategies for managing emotional dysregulation—and allows clinicians to account for the coexistence of distinct intrapsychic perspectives within an individual. As the most psychometrically robust modes, PM and EDO appear to be of particular significance in this population, highlighting the importance of incorporating these modes within individual conceptualisations and treatment plans. For example, while one mode may genuinely strive for recovery and wellbeing, another (e.g., PM) may undermine these efforts by reinforcing beliefs of unworthiness, leading to heightened loneliness and shame (VC). The EDO may attempt to protect against perceived rejection through rigid perfectionism and emotional suppression, which can, in turn, trigger compensatory responses such as impulsive binge and loss of control (IC, DSS). Once an individual’s schema mode profile is identified through the (EN)-SMI-ED-SF, clinicians can more accurately track these mode sequences and dynamics. This enhances therapeutic attunement and allows for targeted interventions that address the specific modes driving the multifaceted aspects of the eating disorder. Experiential ST techniques may be of particular importance in combatting the toxic shame-inducing nature of the PM mode, whilst empathically confronting the cognitive rigidity and intellectualizing tendencies of the EDO. It is only by robustly managing these modes that the individual can begin to relinquish rigid ED behaviours, and shift toward strengthening the HA and developing healthy ways of meeting the emotional needs of the VC mode. Moreover, insight promotes greater self-awareness in individuals with ED, enabling them to understand the historical origins and relational functions of maladaptive modes—often shaped by early unmet emotional needs. Importantly, these results also highlight the importance of strengthening the HA mode in therapy. Fostering self-compassion and psychological flexibility can support the development of more adaptive emotional regulation capacities, particularly when responding to internal experiences of vulnerability (VC). By nurturing the HA mode, individuals may cultivate a more integrated and compassionate sense of self, facilitating meaningful and sustainable recovery trajectories.

## Conclusions

The EN-SMI-ED-SF showed strong psychometric properties, supporting its validity and reliability in assessing schema modes in individuals with eating disorders. The results highlight the relevance of schema modes in explaining the emotional and cognitive mechanisms underlying ED, as well as their links with personality traits. This tool can support clinicians in identifying maladaptive modes and tailoring treatment to individual needs. Its use can enhance case formulation, guide personalized interventions, and help monitor therapeutic progress, promoting more effective and schema-informed care pathways.

## Supplementary Information

Supplementary Material 1

## Data Availability

The datasets presented in this article are not readily available because due to privacy restrictions, data were available from the corresponding author on a reasonable request.
