# Supplementary material for: Assessing schema modes for eating disorders and their association with personality traits: validation of the English version of the short form of the schema modes inventory for eating disorders (EN-SMI-ED-SF)
Source: J Eat Disord. 2025 Nov 11;13:258. doi: 10.1186/s40337-025-01446-0 (PMC12606930; doi:10.1186/s40337-025-01446-0)
Supplement: Supplementary file 1 — Supplementary Material 1 [file 40337_2025_1446_MOESM1_ESM.pdf]

The Schema Modes Inventory for Eating Disorders - Short Form (EN-SMI-ED-SF)

Please answer all the questions by placing a single mark next to the numerical value that best represents how you feel at this moment.

0<br>Never or Almost Never	1<br>Rarely	2<br>Occasionally	3<br>Frequently	4<br>Most of the Time	5<br>All of the Time

1	I feel lonely	0	1	2	3	4	5
2	If I lose control of my eating I feel unsafe	0	1	2	3	4	5
3	I feel lost	0	1	2	3	4	5
4	I feel weak and helpless	0	1	2	3	4	5
5	I have a lot of anger inside me that I can only soothe through my eating behaviors (e.g., restriction, bingeing, purging, exercising)	0	1	2	3	4	5
6	I feel like telling people off for the way they have treated me	0	1	2	3	4	5
7	I have a lot of anger built up inside of me that I need let out	0	1	2	3	4	5
8	I feel like lashing out or hurting someone for what he/she did to me	0	1	2	3	4	5
9	I destroy things when I’m angry	0	1	2	3	4	5
10	I have rage outbursts	0	1	2	3	4	5
11	My anger gets out of control	0	1	2	3	4	5
12	I have been so angry that I emotionally hurt others (*e.g.*, by shouting at him/her)	0	1	2	3	4	5
13	I say what I feel, or do things impulsively, without thinking of the consequences	0	1	2	3	4	5
14	It feels impossible for me to control my impulses	0	1	2	3	4	5
15	I act first and think later	0	1	2	3	4	5
16	If I feel the urge to do something, I just do it	0	1	2	3	4	5
17	I don’t discipline myself to complete routine or boring tasks	0	1	2	3	4	5
18	I can’t bring myself to do things that I find unpleasant, even if I know it is for my own good	0	1	2	3	4	5
19	It’s not worth the effort to plan how you’ll handle situations	0	1	2	3	4	5
20	If I can’t reach a goal, I become easily frustrated and give up	0	1	2	3	4	5
21	I feel loved and accepted	0	1	2	3	4	5
22	I feel at peace on my own	0	1	2	3	4	5
23	I feel content and at ease	0	1	2	3	4	5
24	I feel connected to other people	0	1	2	3	4	5
25	I don't deserve anything that gives me pleasure (*e.g.*, eating, play, nurturance)	0	1	2	3	4	5
26	I’m a bad person	0	1	2	3	4	5
27	I don’t allow myself to do pleasurable things that other people do because I’m bad	0	1	2	3	4	5
28	I deny myself pleasure because I don’t deserve it	0	1	2	3	4	5
29	I demand high standards of my body to avoid being judged	0	1	2	3	4	5
30	I sacrifice pleasure, health, or happiness to meet my own standards	0	1	2	3	4	5

Simpson, Rossi, Mannarini, Tait, Castelnuovo & Pietrabissa et al. (2025). Assessing Schema Modes for Eating Disorders and their Association with Personality Traits: Validation of the English Version of the Short Form of the Schema Modes Inventory for Eating Disorders (EN-SMI-ED-SF). *Journal of eating disorders*.

31	My life revolves around getting things done and doing them right	0	1	2	3	4	5
32	I know that there is a ‘right’ and a ‘wrong’ way to do things; I try hard to do things the right way, or else I start criticising myself	0	1	2	3	4	5
33	I feel that I am basically a good person	0	1	2	3	4	5
34	I assert what I need without going overboard	0	1	2	3	4	5
35	I have a good sense of who I am and what I need to make myself happy	0	1	2	3	4	5
36	I feel able to learn, grow, and change	0	1	2	3	4	5
37	I let other people get their own way instead of expressing my own needs	0	1	2	3	4	5
38	In relationships, I let the other person have the upper hand	0	1	2	3	4	5
39	I try very hard to please other people in order to avoid conflict, confrontation or rejection	0	1	2	3	4	5
40	In relationships, I have to give more to compensate for my lack of worth	0	1	2	3	4	5
41	I feel distant from other people	0	1	2	3	4	5
42	If people try to come too close I keep them at a distance	0	1	2	3	4	5
43	I feel detached (no contact with myself, my emotions or other people)	0	1	2	3	4	5
44	I don’t care about anything; nothing matters to me	0	1	2	3	4	5
45	My eating behaviours (e.g., restriction, bingeing, purging, exercising) help me to detach from difficult emotions	0	1	2	3	4	5
46	I like doing something exciting or soothing to avoid my feelings (e.g., working, gambling, eating, exercise, shopping, sexual activities, watching TV)	0	1	2	3	4	5
47	I work or play sports intensively so that I don’t have to think about upsetting things	0	1	2	3	4	5
48	I want to distract myself from upsetting thoughts and feelings	0	1	2	3	4	5
49	I’m quite critical of other people	0	1	2	3	4	5
50	I feel I shouldn’t have to follow the same rules that other people do	0	1	2	3	4	5
51	Thinness is a way in which I can be better than others	0	1	2	3	4	5
52	I’m demanding of other people	0	1	2	3	4	5
53	By dominating other people, nothing can happen to you	0	1	2	3	4	5
54	I belittle others	0	1	2	3	4	5
55	If you don’t dominate other people, they will dominate you	0	1	2	3	4	5
56	I always look for ways to outsmart others, to ensure that they cannot take advantage of me or hurt me in any way	0	1	2	3	4	5
57	I want people to understand me without me having to say anything	0	1	2	3	4	5
58	I need people to listen to me and make me feel better	0	1	2	3	4	5
59	It’s too hard to make changes to my behavior	0	1	2	3	4	5
60	I feel angry and desperate when people can’t see I need help	0	1	2	3	4	5
61	Feeling in control of my eating ‘trumps’ any problems or disappointments going on in my life	0	1	2	3	4	5
62	Controlling my eating gives me a physical and mental ‘high’	0	1	2	3	4	5
63	Controlling my eating makes me feel in control of everything	0	1	2	3	4	5
64	Controlling my eating stops me being too needy	0	1	2	3	4	5
